# D2 receptor activation modulates NMDA receptor antagonist-enhanced high-frequency oscillations in the olfactory bulb of freely moving rats

**DOI:** 10.1007/s00213-025-06808-9

**Published:** 2025-05-27

**Authors:** Jacek Wróbel, Daniel Krzysztof Wójcik, Mark Jeremy Hunt

**Affiliations:** Laboratory of Neuroinformatics, Nencki Institute of Experimental Biology of Polish Academy of Sciences, 3 Pasteur Street, 02-093 Warsaw, Poland

**Keywords:** Olfactory bulb, NMDA receptor antagonist, MK801, Dopamine receptor, Dopamine antagonist, Dopamine agonist, HFO, Oscillations, Antipsychotics, Local infusion

## Abstract

**Rationale:**

NMDA receptor antagonists, used to model psychotic-like states and treat depression, enhance the power of high-frequency oscillations (HFO) in many mammalian brain regions. In rodents, the olfactory bulb (OB) is a particularly important site for generating this rhythm. OB projection neurons express D1 and D2 receptors (D1R and D2R) which interact with NMDA receptors.

**Objectives:**

The aim of this study was to explore the effect of dopamine (DA) signalling in the OB on MK801-enhanced HFO.

**Methods:**

Local field potentials from the OB and locomotor activity were recorded in adult male freely moving rats. MK801 was injected systemically or infused locally to the OB. The effects of D1R and D2R agonists (SKF38393, quinpirole) and antagonists (SCH23390, eticlopride), administered systemically or locally to the OB, were examined on MK801-enhanced HFO. Effects of the antipsychotics risperidone and aripiprazole were also examined.

**Results:**

Local infusion of MK801 enhanced HFO power in the OB to levels similar to those observed after systemic injection. Neither systemic nor local blockade of D1R or D2R affected the MK801-enhanced HFO, despite reductions in hyperlocomotion. However, direct (systemic and local) D2R, but not D1R, stimulation caused a short-lasting reduction of MK801-enhanced HFO power and longer lasting reduction in frequency. Risperidone, but not aripiprazole, reduced MK801-enhanced HFO frequency.

**Conclusions:**

These results suggest that NMDA receptor antagonist-enhanced HFO in the OB is generated predominantly independently of DA influence, however exogenous stimulation of D2R can modulate this rhythm. A second, but not third generation antipsychotic reduced HFO frequency.

## Introduction

N-methyl-D-aspartate receptor (NMDAR) antagonists produce dissociative states, one of the reasons for their abuse, and are used to model psychotic-like states in humans and rodents (Olney et al. 1999; Ait Bentaleb et al. 2024). Neural oscillations, or brain rhythms, are crucial for synchronising activity across neural circuits and maintaining cognitive functions (Buzsáki et al. 2013). Abnormal brain rhythms occur after the administration of NMDAR antagonists in humans and rodents, and these rhythms have been implicated in psychiatric diseases (Hunt and Kasicki 2013; Nugent et al. 2019; Bianciardi and Uhlhaas 2021; Speers and Bilkey 2021).

Fast oscillations, such as gamma in the cortex, are believed to be associated with higher mental functions (Trimper et al. 2017). NMDAR antagonists are known to increase the power of high-frequency oscillations (> 130 Hz HFO) across many brain regions of mammals. This has been studied primarily in rats (Hunt et al. 2006; Hakami et al. 2009; Hiyoshi et al. 2014; Sokolenko et al. 2019), but has also been observed in mice (Hunt et al. 2015) and cats (Średniawa et al. 2021; Castro-Zaballa et al. 2025), with some evidence suggesting it may also occur in monkeys (Yan et al. 2022) and humans (Nottage et al. 2023). Recent studies have shown that the olfactory bulb (OB) is critical for the generation of HFO after NMDAR blockade which, in turn, is dependent on nasal respiration (Hunt et al. 2019; Wróbel et al. 2020; Średniawa et al. 2021; Castro-Zaballa et al. 2025). The OB is known to powerfully affect rhythms in cortical and subcortical areas (Chen et al. 2023). In line with this, we have shown that reversible inhibition of the OB reduces HFO power in the ventral striatum and prefrontal cortex after ketamine (Hunt et al. 2019; Wróbel et al. 2020), and in the piriform cortex after MK801 (Wróbel et al. 2024). Although the nature of this rhythm is beginning to be elucidated, it remains unclear whether local blockade of NMDAR in the OB is sufficient to generate HFO.

NMDAR and dopamine (DA) D1 and D2 receptors (D1R and D2R) can co-exist in the same neurons and synapses allowing for direct interactions (Lothmann et al. 2021). Co-expression of these receptors allows DA to modulate NMDAR-mediated glutamatergic transmission, influencing synaptic plasticity and neuronal function. This interaction plays a critical role across various brain regions, for example in the striatum, it regulates motor control (Dunah and Standaert 2001) and contributes to reward processing (Lee et al. 2011), while in the frontal cortex, it supports cognitive functions such as decision-making and memory formation (Williams and Castner 2006; Castner and Williams 2007). Blocking DA receptors can reverse certain neurophysiological and behavioural changes induced by NMDAR antagonists. For example, DA antagonists have been shown to reduce the locomotor stimulation caused by MK801 (Ouagazzal and Amalric 1995). Modulatory neurotransmitters, such as DA, are known to influence olfactory circuits (Hsia et al. 1999; Liu 2020; Korshunov et al. 2020; Fischer et al. 2020). The OB is abundant in DA neurons and is thought to represent one of the primary sources of DA neurons in the forebrain of rodents (Björklund and Dunnett 2007). Notably, DA receptors are expressed in mitral/tufted cells and granule cells (Coronas et al. 1997; Gutièrrez-Mecinas et al. 2005), which we have speculated are involved in HFO generation (Hunt et al. 2019; Średniawa et al. 2021). Given the importance of DA in the OB, the primary aim of this study was to examine the role of DA receptors in MK801-enhanced HFO. Additionally, we tested whether the local blockade of NMDAR in the OB is sufficient to increase HFO power.

## Methods

### Surgery

 Adult male Wistar rats (250–350 g) were anaesthetised using isoflurane and placed in a stereotaxic frame. Male rats were used to maintain consistency with our previous research and those of other groups investigating the activity we examined here. All rats (N = 50) were implanted bilaterally with a pair of stainless steel electrodes (125 μm diameter, Science Products, Germany) in the OB (AP + 6.7, ML ± 0.5, DV 3–3.5 mm). Additionally, some rats (N = 36) were implanted bilaterally with 22-gauge stainless steel guides targeting the OB (Bilaney, Germany). A screw posterior to the bregma was used as a reference/ground in all cases.

### Recording

 One week after surgery, rats were placed in an arena (44 × 50 × 42 cm). Local field potentials (LFP) were recorded through a JFET preamplifier, amplified 1000 ×, filtered 0.1–1000 Hz (A-M Systems, USA), and digitised at 5 kHz (Micro1401, CED, Cambridge, UK). When possible horizontal locomotor activity (LMA) was assessed by photocell beam breaks (Columbus Instruments, USA). Rats were recorded for 2 days prior to the main experiment to habituate them to the recording chamber.

### Systemic experiments

 Rats were recorded for 20 min. and then injected (i.p.) with 0.15 mg/kg MK801 (Sigma, Poland) followed, 25–30 min. later by injection of: group 1 (N = 7): 2 mg/kg amphetamine, 2 mg/kg apomorphine, or control saline; group 2 (N = 7): 1 mg/kg quinpirole or 1 mg/kg SKF38393; or group 3 (N = 7 rats): 1 mg/kg eticlopride + 1 mg/kg SCH23390, 3 mg/kg risperidone, 3 mg/kg aripiprazole and control saline or dimethyl sulfoxide. Rats were injected with drugs in a pseudo randomised order so that each rat in a particular group received an injection of all drugs or vehicles.

### Bilateral MK801 infusion experiments (N = 7)

 Briefly, 20 min. after recording a 28 gauge infusion needle (Bilaney, Germany) was inserted and descended 1 mm below the tip of the guide implanted in the OB. Infusions were carried out at a rate of 0.5 µl/min. for 1 min. MK801 or saline (0.5 µl) was infused to the OB.

### Bilateral DA agonist/antagonist infusion experiments (N = 29)

 Rats were recorded for 20 min. Briefly, 30 min. post i.p. injection of 0.15 mg/kg MK801 a 28 gauge infusion needles (Bilaney, Germany) was inserted bilaterally and descended 1 mm below the tip of the guide implanted in the OB. Infusions were carried out at a rate of 0.5 µl/min. for 1 min. DA agents (Sigma, Poland) or saline (0.5 µl) were infused to the OB. Four separate groups of rats were used. Each group received an infusion of saline and only one agonist or antagonist at two doses: D1R agonist SKF38393 at 2.5 µg/side or 5 µg/side (N = 7); D2R agonist quinpirole at 2.5 µg/side or 12.5 µg/side (N = 8); D1R antagonist SCH23390 at 1 µg/side or 6 µg/side (N = 7); or D2R antagonist eticlopride at 2.5 µg/side or 12.5 µg/side (N = 7).

### Drugs

 MK801, amphetamine, apomorphine, SKF38393, SKF83566-green, quinpirole, SCH23390 and eticlopride were dissolved in saline whereas risperidone and aripiprazole in dimethyl sulfoxide.

### Histology

 At the end of the study rats were terminated. Brains were fixed using 4% paraformaldehyde (Sigma, Poland) and preserved using 30% sucrose (Sigma, Poland). Electrolytic lesions were made for the implanted rats and electrode locations were determined on 35 µm Cresyl violet (Sigma, UK) stained sections.

### Data analyses

**Fig. 1:**
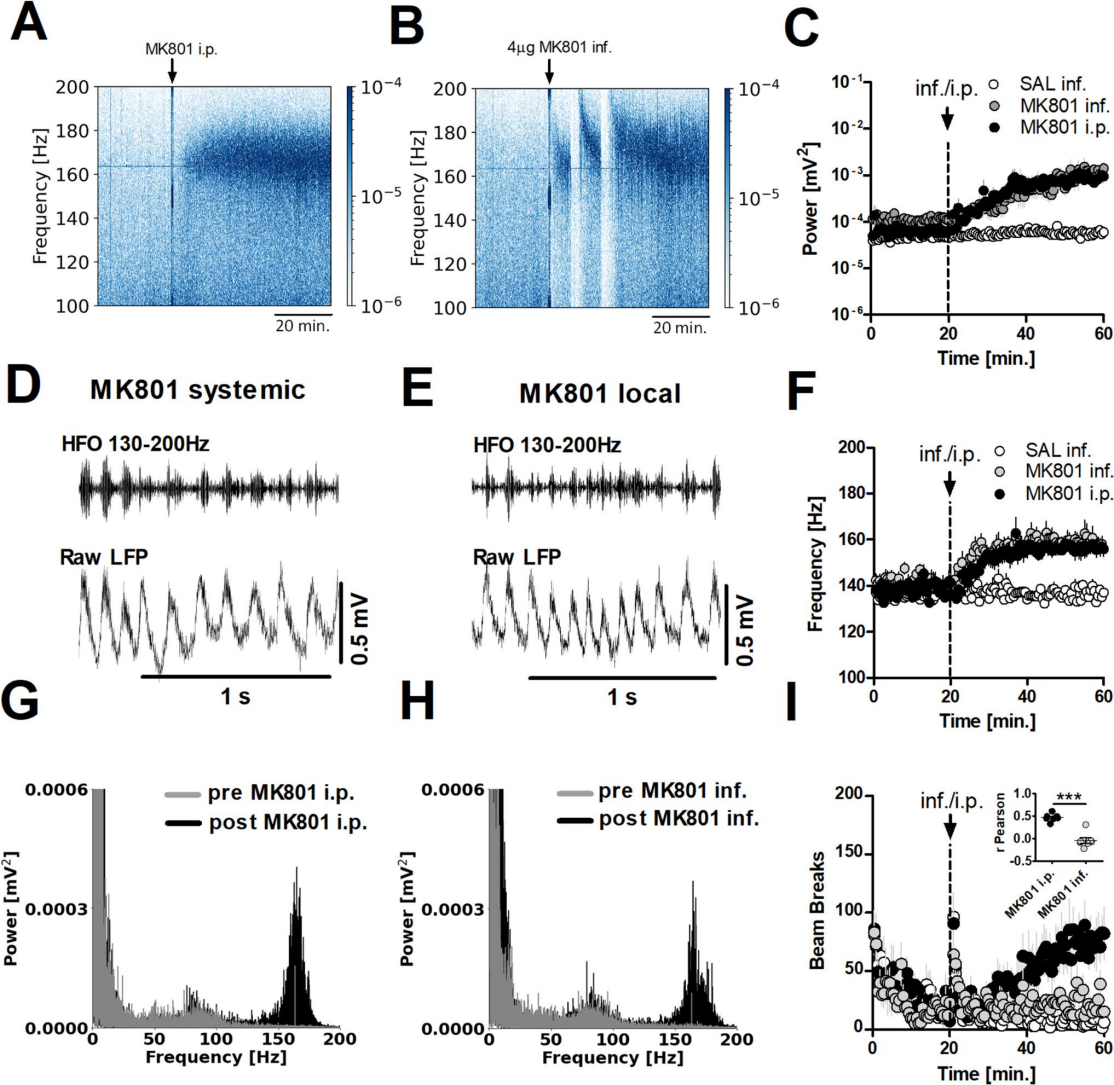
Systemic and local blockade of NMDAR in the OB increase HFO power but differentially affect LMA. **A** Example spectrograms showing MK801-enhanced HFO after 0.15 mg/kg i.p. or (**B**) 4 μg/side local infusion. **C** Complete time-course showing the changes in power after systemic and local infusion of MK801 or saline (*N* = 7). 2-way ANOVA revealed a significant time × group interaction (*p* < 0.0001). Bonferroni’s post hoc test revealed a significant effect for saline inf. vs MK801 inf., *p* < 0.001 and saline inf. vs MK801 i.p., *p* < 0.01. There was no significant difference between MK801 inf. vs MK801 i.p. **D** Example raw and band-pass filtered (130–200 Hz) signals after 0.15 mg/kg MK801 i.p. injection or (**E**) 4 μg/side OB infusion of MK801 recorded in the OB. Note HFO are located in bursts in both cases. **F** Complete time-course showing the changes in frequency after MK801 i.p. injection (*N* = 7). 2-way ANOVA revealed a significant time × group interaction (*p* < 0.0001). Bonferroni’s post hoc test revealed an effect for saline vs MK801 inf. and saline vs MK801 i.p., *p* < 0.0001). There was no difference between MK801 inf. vs MK801 i.p. **G** Power spectra (60 s) before and after MK801 i.p. injection or (**H**) OB bilateral infusion of MK801 presenting a HFO peak around 160 Hz. **I** Complete time-course presenting LMA after MK801 i.p. injection, MK801 OB bilateral infusion and saline OB bilateral infusion (*N* = 7). 2-way ANOVA revealed a significant time × group interaction (*p* < 0.0001). Bonferroni’s post hoc test revealed an effect for saline vs MK801 i.p. and MK801 i.p. vs MK801 inf., *p* < 0.0001). There was no difference between saline vs MK801 inf. Scatter dot plot shows comparison between correlation of LMA and HFO power after MK801 injection and infusion. LMA showed a stronger correlation with HFO power following MK801 administered via injection (i.p.) compared to MK801 infusion (Pearson r value for MK801 i.p. = 0.46 ± 0.031, for MK801 inf. = −0.05 ± 0.062, paired t-test, *p* = 0.0004). ***p < 0.001

**Fig. 2:**
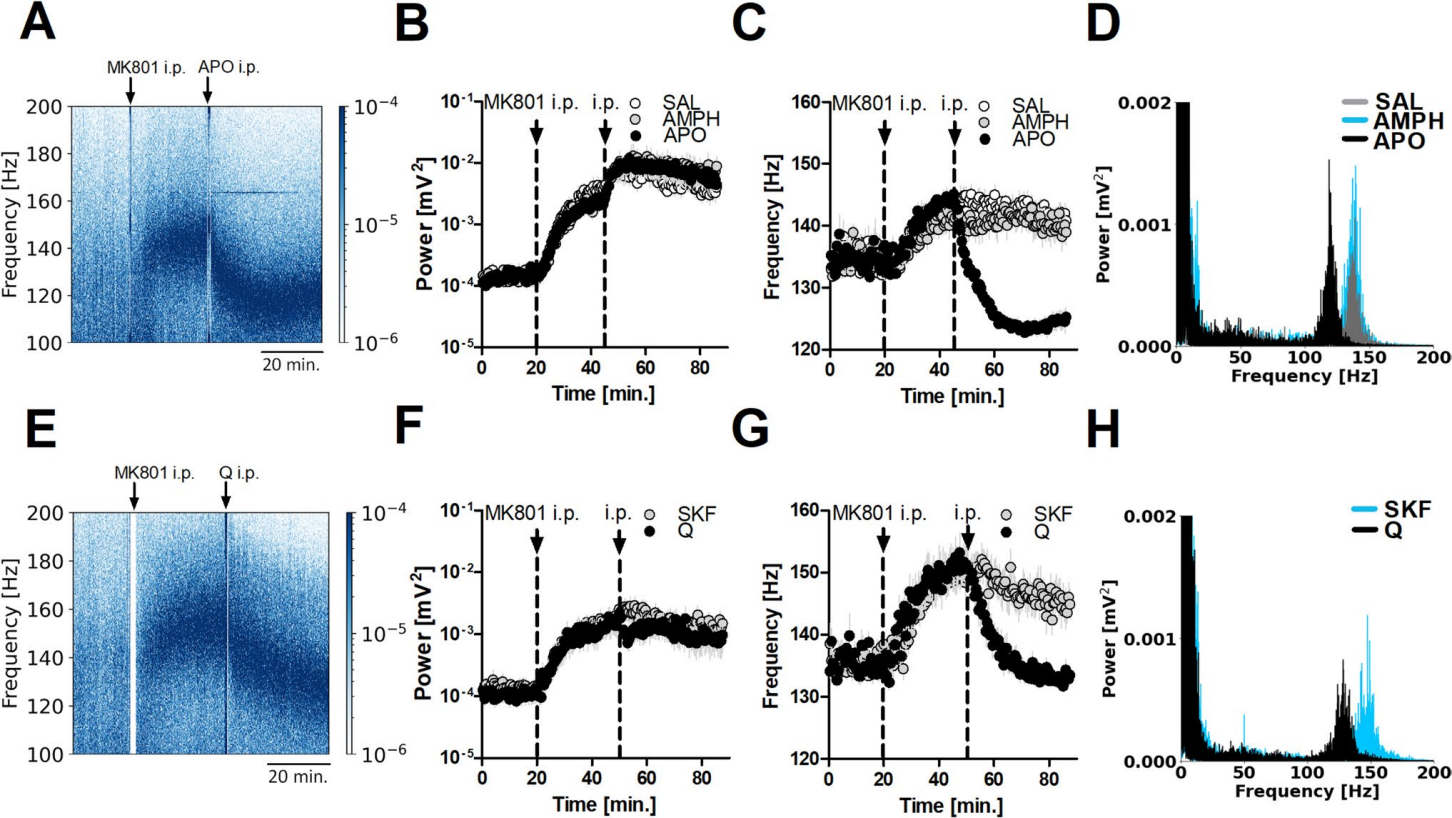
Systemic stimulation of DA receptors reduces MK801-enhanced HFO frequency in the OB via a mechanism involving D2R. **A** Example spectrogram showing the effect of 2 mg/kg systemic apomorphine (APO) on MK801-enhanced HFO. **B**, **C** Complete time-courses presenting effects of 2 mg/kg amphetamine (AMPH) and APO on power and frequency of MK801-enhanced HFO in the OB (N = 7). For power, repeated measures 2-way ANOVA revealed no significant interaction. For frequency, repeated measures 2-way ANOVA revealed a significant time × group interaction (p < 0.0001). Bonferroni’s post hoc test revealed an effect for MK801 + saline vs MK801 + APO and MK801 + APO vs MK801 + AMPH, *p* < 0.0001 and no significant effect for MK801 + saline vs MK801 + AMPH. **D** Power spectra (60 s) showing HFO frequency peak after systemic injection of APO (black), AMPH (blue) and saline (grey). Note HFO frequency peak after APO is shifted to lower frequencies. **E** Example spectrogram showing the effect of 1 mg/kg systemic quinpirole (Q) on MK801-enhanced HFO. **F, G** Complete time-courses presenting effects of Q and 1 mg/kg SKF38393 (SKF) on power and frequency of MK801-enhanced HFO in the OB (*N* = 7). For power, repeated measures 2-way ANOVA revealed no significant time × group interaction (p = 0.093). For frequency, repeated measures 2-way ANOVA revealed a significant time × group interaction (p < 0.0001). Bonferroni’s post hoc test revealed an effect for MK801 + Q vs MK801 + SKF, *p* < 0.01). **H** Power spectra (60 s) showing HFO frequency peak after systemic injection of Q (black) and SKF (blue). Note HFO frequency peak after Q is shifted to lower frequencies

**Fig. 3:**
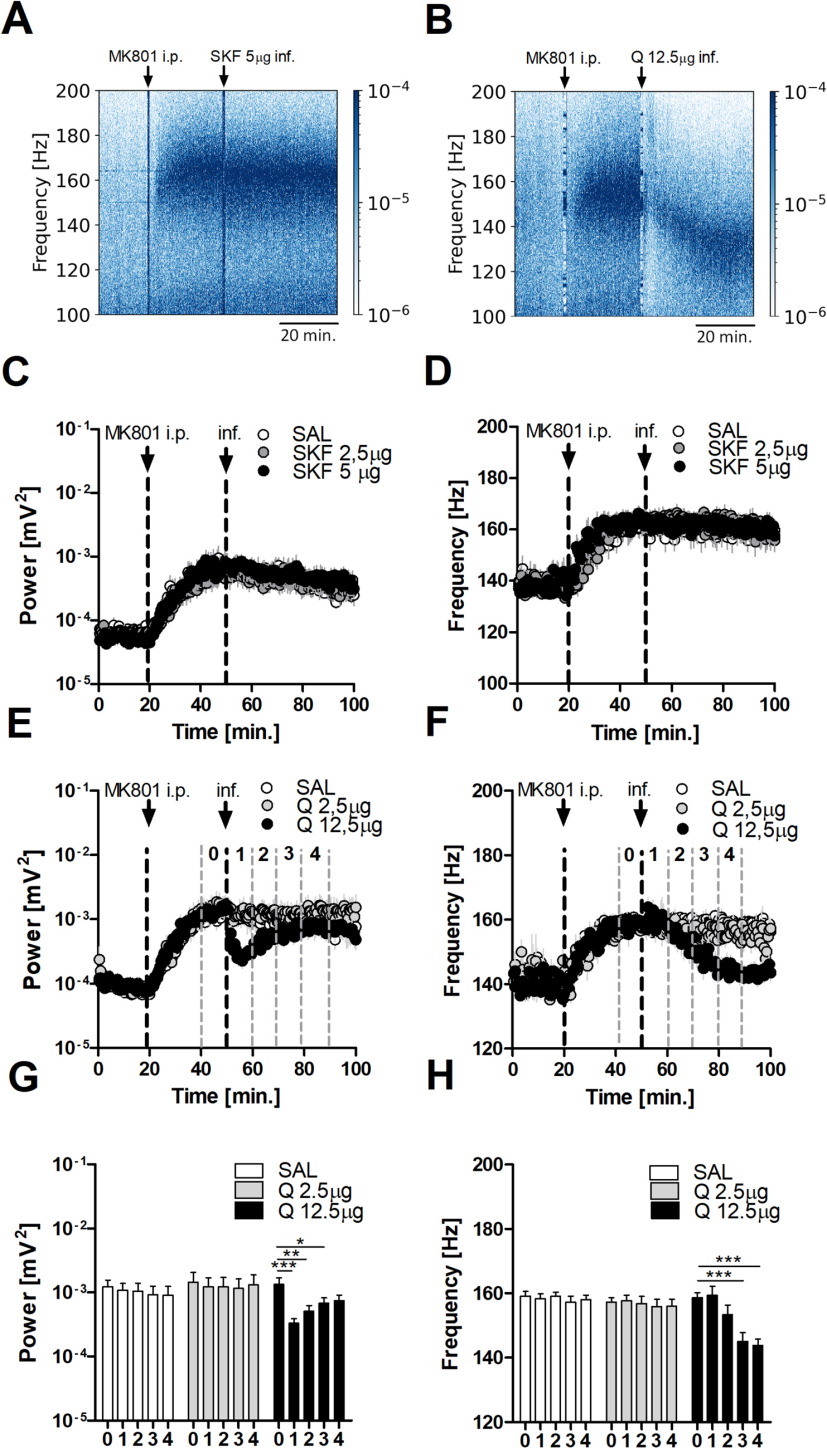
Local OB infusion of D1R & D2R agonists differentially affects MK801-enhanced HFO in the OB. **A**, **B** Spectrograms showing MK801-enhanced HFO after 5 µg/side D1R agonist SKF38393 (SKF) and 12.5 µg/side D2R agonist quinpirole (Q). **C, D** Complete time-courses presenting effect of 2.5 and 5 µg/side SKF (*N* = 7) on power and frequency of MK801-enhanced HFO. Repeated measures 2-way ANOVA revealed no significant time × group interaction (*p* = 1.000 for power, p = 0.573 for frequency). **E, F** Complete time-courses presenting effect of 2.5 and 12.5 µg/side Q (*N* = 8) on power and frequency of MK801-enhanced HFO. The dashed lines represent 10-min. time bins before and after infusion of Q. **G, H** Bar charts showing power and frequency of MK801-enhanced HFO after 2.5 and 12.5 µg/side Q infusion for the 10-min. bins shown in E and F. Repeated measures 1-way ANOVA revealed no significant effect for SAL and Q 2.5 inf. (*p* < 0.05 for power and frequency) but a significant effect for Q 12.5 inf. (*p* = 0.0007 for power, *p* < 0.0001 for frequency); *p < 0.05, **p < 0.01, ***p < 0.001 (Bonferroni’s post hoc test)

 LFP recordings stored in Spike2 format were imported into Python for analysis. Signal processing was performed using the SciPy Signal and NumPy libraries, focusing on bandpass filtering with Butterworth filters. Narrowband noise was removed using additional notch filters when necessary. Spectrogram analysis, based on the Short-Time Fourier Transform, was applied to 30 s windows to assess the power and dominant frequency in specific frequency ranges. For the analysis of HFO, we used a frequency band of 130–200 Hz in all experiments, except for the apomorphine i.p. injection experiment (Fig. 2C). In that specific case, a 120–200 Hz range was employed to capture the reduction in HFO frequency below 130 Hz. For analysis of HFO power and frequency after quinpirole local infusion (Fig. 3G and H) LFP were divided in 5 batches (fragment 0 – pre infusion, fragments 1–4 post infusion, each fragment = 10 min.). For comparison between frequencies in Fig. 1 and Fig. 2, power spectra were computed from 60-s segments, taken 40 min. after infusion or injection.

### Statistics and data visualization

 All groups were tested using the Shapiro–Wilk’s test for normality. For the analysis we used a 1 or 2-way ANOVA followed by Bonferroni’s post hoc test or paired Student’s t-test. In all cases, differences were considered significant when p ≤ 0.05. Data were anaylzed using GraphPad Prism (GraphPad Software, San Diego, USA). Data are presented as mean ± SEM, unless stated otherwise.

## Results

### Systemic and local blockade of NMDAR in the OB increase HFO power but differentially affect LMA

We investigated the effect of systemic injection (i.p.) of 0.15 mg/kg MK801 and 4 μg/side intra-OB infusion in 7 rats. Example spectrograms showing MK801-enhanced HFO after i.p. or local infusion are shown in Fig. 1A and B, respectively. In both cases administration was associated with an increase in the power of HFO. Examples of raw LFP and 130–200 Hz band-pass filtered signals after systemic injection or local infusion of MK801 are shown in Fig. 1D and E, respectively. Notably, after local infusion, but not systemic injection, we observed occasional short-lasting (50–200 s) attenuation of HFO, which was followed by recovery of HFO at a higher frequency. Representative power spectra for 60 s before and after MK801 administration are shown in Fig. 1G and H, respectively, and were associated with increases in the HFO band. Complete time-courses showing increases in HFO power (Fig. 1C) and frequency (Fig. 1F) were comparable for systemic and local infusion of MK801. Repeated measures 2-way ANOVA revealed a significant time × group interaction for HFO power (F_(318,2862)_ = 2.76, *p* < 0.0001) and HFO frequency (F_(318,2862)_ = 4.22, *p* < 0.0001). In both cases Bonferroni’s post hoc test revealed a significant effect for power (saline vs MK801 infusion, p < 0.001; saline vs MK801 i.p., *p* < 0.01) and frequency (saline vs MK801 infusion; saline vs MK801 i.p., *p* < 0.0001). There was no significant difference between MK801 infusion vs MK801 i.p., *p* > 0.05.

Despite similar increases in HFO power, LMA increased after systemic MK801 administration (i.p.), but not after local infusion (Fig. 1I). Repeated measures 2-way ANOVA revealed a significant time × group interaction (F_(318,2862)_ = 3.47, *p* < 0.0001). Bonferroni’s post hoc test revealed a significant effect for saline vs MK801 i.p. and MK801 i.p. vs MK801 infusion, *p* < 0.0001. There was no significant difference between saline vs MK801 inf., *p* > 0.05. Changes in LMA correlated with HFO power after MK801 i.p. in 7/7 rats, but only 2/7 rats after local infusion. Pearson r values for MK801 i.p. = 0.46 ± 0.03 vs MK801 infusion = −0.05 ± 0.06 were significantly different (paired t-test, p = 0.0004; Fig. 1I, insert).

### Systemic stimulation of DA receptors reduces MK801-enhanced HFO frequency in the OB *via* a mechanism involving D2R

Having shown that local blockade of NMDAR in the OB alone is sufficient to increase HFO power, and considering the complex interactions between NMDAR, D1R, and D2R (Lothmann et al. 2021), we next examined the effects of DA agonists and antagonists on this rhythm. First, we studied the effect of systemic administration of amphetamine and apomorphine on MK801-enhanced HFO (*N* = 7). Amphetamine is an indirect non-selective agonist of DA receptors which increases the amount of DA (and other monoamines) in the synaptic cleft through various mechanisms, such as increasing DA release from the presynaptic terminal, inhibiting DA reuptake (Faraone 2018). Apomorphine stimulates both D1R and D2R directly and is a direct, non-selective agonist of DA receptors (Jenner and Katzenschlager 2016). An example spectrogram showing the effect of 2 mg/kg systemic apomorphine injection on MK801-enhanced is shown in Fig. 2A. Neither amphetamine nor apomorphine affected the power of MK801-enhanced HFO (Fig. 2B). Repeated measures 2-way ANOVA revealed no significant time × group interaction (F_(338,3042)_ = 0.69, *p* = 1.000). Other studies have shown that amphetamine does not markedly affect HFO power (Hunt et al. 2006; Hansen et al. 2019; Brys et al. 2023). Administration of apomorphine, but not amphetamine, reduced the frequency of MK801-enhanced HFO (Fig. 2C). The mean frequency 40 min. post injection was 125 ± 0.35 Hz for apomorphine vs 141 ± 0.58 Hz for saline. Repeated measures 2-way ANOVA revealed a significant time × group interaction (F_(338,3042)_ = 17.35, *p* < 0.0001). Bonferroni’s post hoc test revealed a significant effect for apomorphine vs saline and apomorphine vs amphetamine, p < 0.0001. No significant differences were found for saline vs amphetamine, *p* > 0.05).

In a separate group of rats (*N* = 7) we tested the effect of a D1R agonist 1 mg/kg SKF38393 and D2R agonist 1 mg/kg quinpirole to determine if a specific DA receptor may account for the change in HFO frequency after MK801. An example spectrogram showing the effect of 1 mg/kg systemic quinpirole injection on MK801-enhanced HFO is shown in Fig. 2E. Time-courses showing the effects of both agonists on MK801-enhanced HFO power and frequency are shown in Fig. 2F and G, respectively. Consistent with results for apomorphine neither selective agonists affected HFO power (time × group interaction (F_(172,2064)_ = 1.15, *p* = 0.093). However, quinpirole significantly reduced MK801-enhanced HFO frequency compared with SKF38393 (Fig. 2G). Repeated measures 2-way ANOVA revealed a significant time × group interaction (F_(172,2064)_ = 7.36, *p* < 0.0001, Bonferroni’s post hoc test *p* < 0.01). The mean frequency 40 min. post injection of quinpirole was 134 ± 0.53 Hz vs 144 ± 0.34 Hz for SKF38393. Figure 2D, H show power spectra with the HFO frequency peak after injection of all drugs. Note that the HFO frequency peak after apomorphine and quinpirole is shifted to lower frequencies.

### Local stimulation of D2R but not D1R affects MK801-enhanced HFO

Given that systemic injection of DA agonists affect MK801-enhanced HFO recorded in the OB, we next examined the effect of local DA receptor stimulation within the OB on HFO. Separate groups of rats were used for both studies; D1R agonist SKF38393 at 2.5 or 5 µg/side (*N* = 7) and D2R agonist quinpirole at 2.5 or 12.5 µg/side (*N* = 8) (Fig. 3). Representative spectrograms for the higher doses of SKF38393 and quinpirole are presented in Fig. 3A and B. Consistent with systemic injections, local infusions of D1R agonist SKF38393 did not substantially affect the power and frequency of MK801-enhanced HFO (Fig. 3C and D). Repeated measures 2-way ANOVA revealed no significant time × group interaction for SKF38393 (F_(438,3942)_ = 0.63, *p* = 1.000 for power; F_(__438,3942__)_ = 0.99, *p* = 0.573 for frequency). Local infusion of quinpirole dose-dependently reduced the power and frequency of MK801-enhanced HFO but with different temporal dynamics. Time-courses showing the effects of quinpirole infusion on MK801-enhanced HFO power and frequency are shown in Fig. 3E and F, respectively. 2-way ANOVA of the complete time-course revealed a significant effect for HFO frequency alone (time × group interaction F_(428,4494)_ = 2.86, *p* < 0.0001), but not power (F_(428,4494)_ = 1.07, *p* = 0.151). Closer inspection of the time-courses revealed an apparent reduction in HFO power associated with local infusion of the D2R agonist. We therefore analysed the data over 5 bins (fragment 0 – pre infusion, fragments 1–4 post infusion, each fragment = 10 min.). Repeated measures 1-way ANOVA revealed effects for both power and frequency (*p* = 0.0007 for power, *p* < 0.0001 for frequency). Bonferroni’s post hoc test revealed a significant effect of 12.5 µg quinpirole infusion for power (*p* < 0.001) and frequency (p < 0.0001) (see Fig. 3G, H). To determine the potential spread of drug to regions outside the OB we infused a separate group of rats with fluorescent SKF83566-green and processed histology across the frontal regions. Supplementary 1 shows the spread of SKF83566-green in the OB 15 min. after infusion, and shows the infusion was relatively well-localized to this region.

### Blockade of DA receptors does not affect MK801-enhanced HFO but reduces LMA

**Fig. 4:**
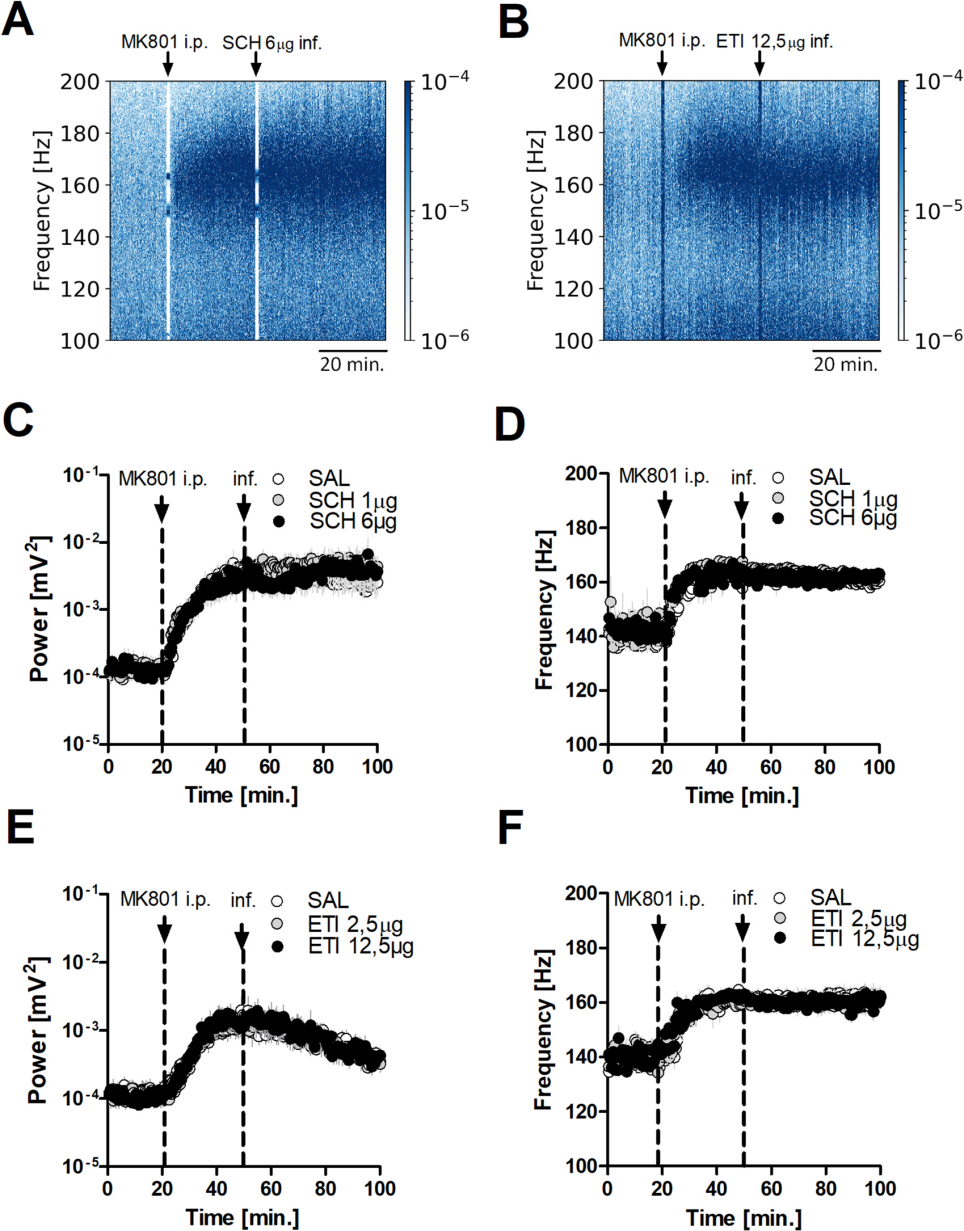
Local OB infusion of D1R & D2R antagonists does not affect MK801-enhanced HFO in the OB. **A**, **B** Spectrograms showing MK801-enhanced HFO after 6 µg/side D1R antagonist SCH23390 (SCH) and 12.5 µg/side D2R antagonist eticlopride (ETI). **C, D** Complete time-courses presenting effect of 1 and 6 µg/side SCH on power and frequency of MK801-enhanced HFO (*N* = 7). Repeated measures 2-way ANOVA revealed no significant time × group interaction (*p* = 1.000 both for power and frequency). **E, F** Complete time-courses presenting effect of 2.5 and 12.5 µg/side ETI on power and frequency of MK801-enhanced HFO (*N* = 7). Repeated measures 2-way ANOVA revealed no significant time × group interaction (*p* = 1.000 for power, *p* = 0.999 for frequency)

Having shown local D2R stimulation can influence the HFO rhythm in the OB, we next tested the effects D1R and D2R antagonists on HFO power and frequency in a separate groups of rats, hypothesising that these might produce effects opposite to the agonists. We tested local infusion of the DA antagonists SCH23390 at 1 or 6 µg/side (*N* = 7), eticlopride at 2.5 or 12.5 µg/side (*N* = 7) (Fig. 4). Representative spectrograms for the higher doses of SCH23390 and eticlopride are presented in Fig. 4A and B. Time-courses showing the effects of SCH23390 and eticlopride infusion on MK801-enhanced HFO power and frequency are shown in Fig. 4C-F. For power repeated measures 2-way ANOVA revealed no significant time × group interaction for SCH23390 (F_(438,3942)_ = 0.74, *p* = 1.000) and for eticlopride (F_(438,3942)_ = 0.51, *p* = 1.000). Infusion of the DA antagonists had also no effects on HFO frequency (SCH23390, F_(438,3942)_ = 0.70, *p* = 1.000; eticlopride; F_(438,3942)_ = 0.79, *p* = 0.999). Although there were no obvious effects on the HFO rhythm, local infusions of both D1R and D2R antagonists dose-dependently reduced MK801-enhanced LMA (see Table 1 for full details).

**Table 1:** Effect of DA antagonists and agonists on MK801-enhanced locomotion

	RECEPTOR	DRUG & DOSES	ANOVA	BONFERRONI POST TEST	SIGNIFICANCE
ANTAGONIST	D1R	SCH23390 1µg, 6µg	p<0.0071	sal vs 1µg	ns
				sal vs 6µg	p<0.05
				1µg vs 6µg	p<0.05
	D2R	eticlopride 2.5µg, 12.5µg	p<0.0287	sal vs 2.5µg	ns
				sal vs 12.5µg	p<0.05
				2.5µg vs 12.5µg	ns
AGONIST	D1R	SKF38393 2.5µg, 5µg	p=0.1280	sal vs 2.5µg	ns
				sal vs 5µg	ns
				2.5µg vs 5µg	ns
	D2R	quinpirole 2.5µg, 12.5µg	p=0.1255	sal vs 2.5µg	ns
				sal vs 12.5µg	ns
				2.5µg vs 12.5µg	ns

Repeated measures 1-way ANOVA of beam break activity revealed significant effects for DA antagonists (p = 0.007 for SCH23390, p = 0.029 for eticlopride); *p < 0.05, Bonferroni’s post hoc test. Repeated measures 1-way ANOVA revealed no change in beam break activity for the DA agonists (p = 0.128 for SKF38393, p = 0.126 for quinpirole).

**Fig. 5:**
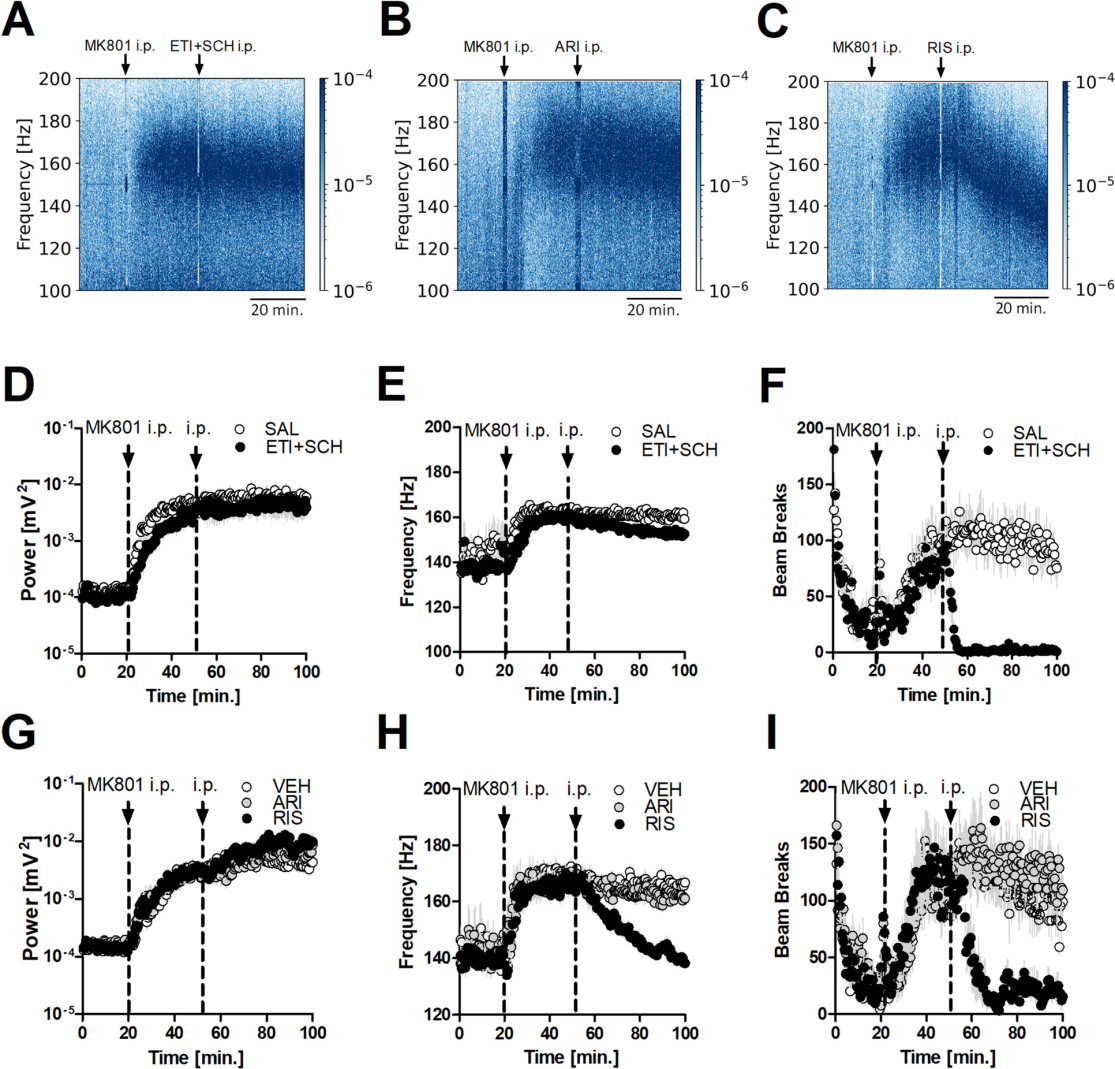
Effect of systemic blockade of D1R and D2R and antipsychotics on HFO power, frequency and LMA. (**A**-**C**) Representative spectrograms illustrating the effects of systemic injection of 1 mg/kg eticlopride + 1 mg/kg SCH23390 (ETI + SCH), 3 mg/kg aripiprazole (ARI), and 3 mg/kg risperidone (RIS) on MK801-enhanced HFO. **D** Complete time-course presenting the effect of coadministration of ETI + SCH on MK801-enhanced power. Repeated measures 2-way ANOVA revealed no significant time × group interaction (*p* = 0.362). **E** Complete time-course presenting the effect of coadministration of ETI + SCH on MK801-enhanced frequency. Repeated measures 2-way ANOVA revealed no significant time × group interaction (*p* = 0.732). **F** Complete time-course presenting the effect of coadministration of ETI + SCH on MK801-enhanced LMA. 2-way repeated measures ANOVA revealed a significant time × group interaction (*p* < 0.0001); Bonferroni’s post hoc test *p* < 0.0001. **G** Complete time-course presenting the effect of systemic ARI, RIS and vehicle dimethyl sulfoxide (VEH) on MK801-enhanced power. Repeated measures 2-way ANOVA revealed a significant effect of time × group interaction (*p* < 0.001). Bonferroni’s post hoc test revealed an effect for VEH vs RIS, *p* < 0.001 and ARI vs RIS, p < 0.05, but no difference for VEH vs ARI. **H** Complete time-course presenting the effect of systemic ARI, RIS and VEH on MK801-enhanced frequency. Repeated measures 2-way ANOVA revealed significant time × group interaction (*p* < 0.0001). Bonferroni’s post hoc test revealed an effect for VEH vs RIS, and ARI vs RIS, both *p* < 0.0001, but no significant difference for VEH vs ARI. (**I**) Complete time-course presenting the effect of systemic ARI, RIS and VEH on MK801-enhanced LMA. Repeated measures 2-way ANOVA revealed significant time × group interaction (*p* < 0.0001). Bonferroni’s post hoc test revealed an effect for VEH vs RIS, *p* < 0.001 and ARI vs RIS, *p* < 0.0001, but no difference for VEH vs ARI

To test whether DA antagonists could affect HFO by affecting regions outside the OB we carried out a separate study using systemic injection of the same antagonists. Rats (N = 7) received co-administration of 1 mg/kg D2R antagonist eticlopride and 1 mg/kg D1R antagonist SCH23390 following 0.15 mg/kg MK801 (Fig. 5). Figure 5A shows an example spectrogram showing the effect of systemic coadministration of eticlopride + SCH23390 on MK801-enhanced HFO. Complete time-courses for HFO power, frequency and LMA are shown in Fig. 5D-F. Repeated measures 2-way ANOVA revealed no significant time × group interaction (F_(219,3066)_ = 1.03, *p* = 0.362 for power, F_(219,3066)_ = 0.94, *p* = 0.732 for frequency), but a significant time × group interaction (F_(220,2640)_ = 9.17, *p* < 0.0001 for LMA (Bonferroni’s post hoc test *p* < 0.0001).

In order to explore the effects of antipsychotics on MK801-enhanced HFO in the OB, we examined the impact of two antipsychotic drugs: aripiprazole, a third-generation antipsychotic which act as a partial agonist at D2R (Bymaster et al. 1996; Wood et al. 2006) and risperidone, a second-generation antipsychotic shown previously to reduce HFO frequency in other brain regions (Olszewski et al. 2013; Delgado-Sallent et al. 2022). Representative spectrograms illustrating the effects of 3 mg/kg aripiprazole and 3 mg/kg risperidone systemic injections on MK801-enhanced HFO are presented in Fig. 5B and C. Time-courses showing the effect of aripiprazole, risperidone and vehicle dimethyl sulfoxide on MK801-enhanced HFO power, frequency and LMA (*N* = 7) are shown in Fig. 5G-I. Repeated measures 2-way ANOVA revealed a significant time × group interaction for power (F_(438,3942)_ = 1.90, p < 0.001), frequency (F_(438,3942)_ = 4.38, *p* < 0.0001) and LMA (F_(438,3942)_ = 4.61, *p* < 0.0001). Bonferroni’s post hoc test revealed a significant effect for power (vehicle vs risperidone, p < 0.001 and aripiprazole vs risperidone, *p* < 0.05), frequency (vehicle vs risperidone and aripiprazole vs risperidone, both *p* < 0.0001) and LMA (vehicle vs risperidone, p < 0.001 and aripiprazole vs risperidone, both *p* < 0.0001), but no difference between vehicle vs aripiprazole, *p* > 0.05, for power, frequency and LMA. This is consistent with other studies showing aripiprazole does not affect MK801-enhanced LMA (Su et al. 2007; Adraoui et al. 2024).

## Discussion

### Local OB networks can mediate HFO

Local infusion of MK801 to the OB increased the power of HFO to levels similar to those observed after systemic injection, indicating local blockade of NMDAR within the OB is sufficient to enhance this rhythm. Increases in HFO power have also been reported following local infusion of MK801 to other brain regions including the nucleus accumbens, prefrontal cortex, and hippocampus (Hunt et al. 2010; Lee et al. 2017). This suggests the network(s) generating this type of HFO could involve widespread brain regions. However, it is worth pointing out that in the study by Lee, relatively large amounts of MK801 were infused (up to 25 μg/side vs up to 4 μg/side in the present study) and therefore actions outside the target infusion area may contribute to the effect observed. Notwithstanding OB activity appears to be crucial for HFO after NMDAR antagonists. For example, HFO recorded in the piriform cortex (Wróbel et al. 2024), and ventral striatum (Hunt et al. 2019; Wróbel et al. 2020), are reduced by pharmacological OB inactivation and blockade of nasal respiration (Hunt et al. 2019; Wróbel et al. 2020, 2024; Średniawa et al. 2021).

How NMDAR blockers generate HFO in the OB is currently unknown, however possible clues may be found from other brain regions. For example, in cortical areas NMDAR antagonists preferentially target constantly depolarizing neurons, particularly parvalbumin-expressing fast-spiking GABAergic interneurons (Homayoun and Moghaddam 2007). This action leads to a reduction in excitatory glutamatergic inputs to these interneurons, resulting in their disinhibition. This mechanism, which has been widely observed across various brain regions, is thought to underlie the disinhibition of projection neurons (Olney et al. 1991; Benes 2000; Lisman et al. 2008). It is plausible that the same holds true in the OB. Indeed, we showed previously that the firing of mitral/tufted cells can phase-lock to the HFO rhythm after ketamine (Hunt et al. 2019). The relationship between HFO and firing of inhibitory interneurons in the OB is currently unknown, and further studies examining their possible role in HFO rhythmogenesis is warranted.

### HFO in the OB and its modulation by D2R

**Fig. 6:**
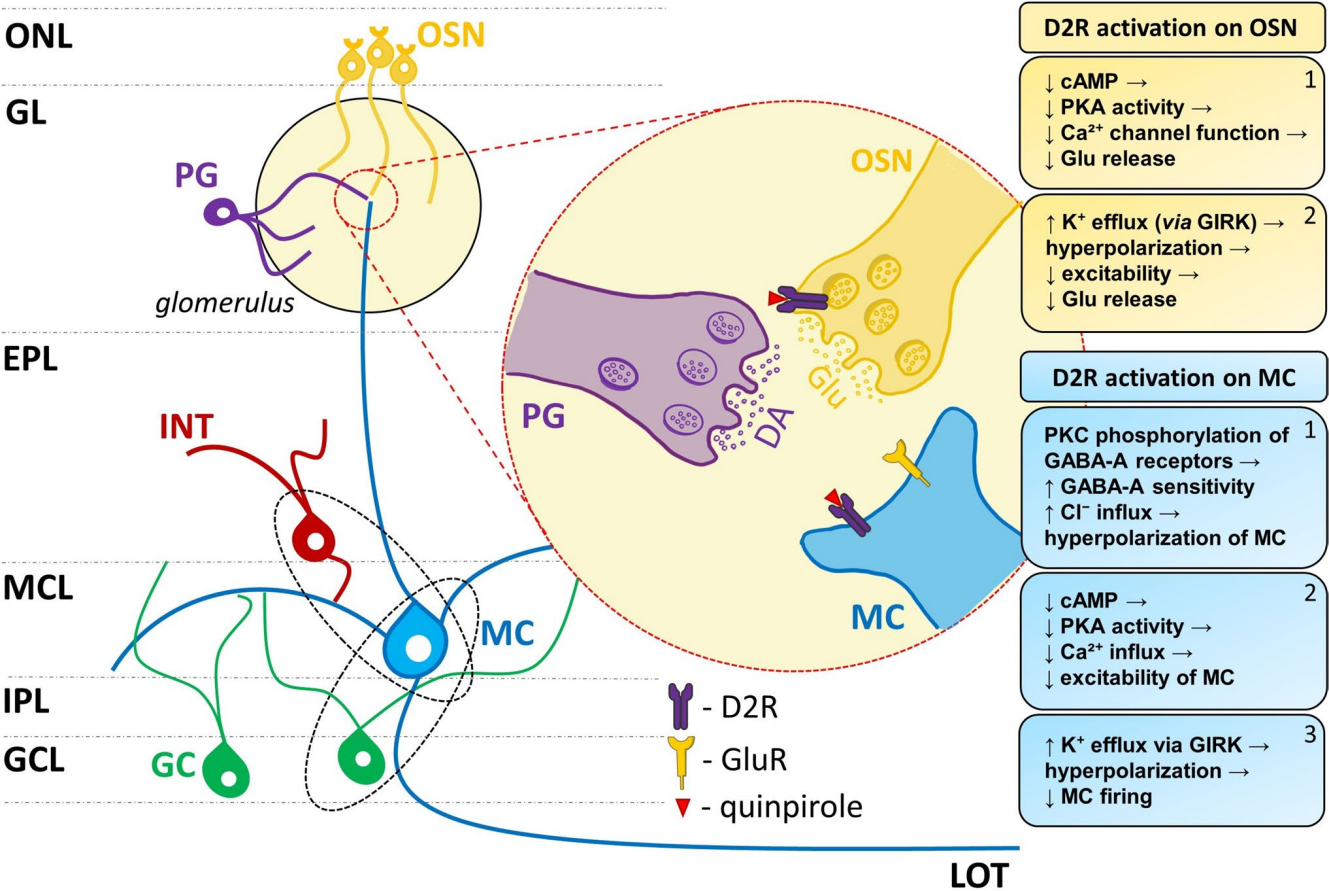
Potential mechanisms through which D2R activation in the OB could modulate MK801-enhanced HFO. Stimulation of D2R on olfactory sensory neuron (OSN) terminals and mitral cell (MC) apical dendrites reduces the excitability of MC. Effects on OSN (yellow): 1) D2R activation decreases cAMP levels, reducing PKA activity and weakening Ca^2^⁺ channel function, which lowers Ca^2^⁺ influx and glutamate (Glu) release from OSN terminals onto the apical dendrites of mitral cells; 2) D2R activation on OSN terminals enhances K⁺ efflux through GIRK channels, causing hyperpolarization and further suppressing Glu release. Effects on MC (blue): 1) D2R activation increases PKC-mediated phosphorylation of GABA-A receptors, enhancing Cl⁻ influx and hyperpolarizing mitral cell membranes; 2) Reduced cAMP levels lower PKA activity, decreasing Ca^2^⁺ influx and the excitability of mitral cells; 3) D2R activation also facilitates K⁺ efflux via GIRK channels, further hyperpolarizing MC. Legend: Ca^2^⁺ – calcium, cAMP – cyclic adenosine monophosphate, Cl⁻ – chloride, DA – dopamine, D2R – dopamine D2 receptor, EPL – external plexiform layer, GABA – gamma-aminobutyric acid, GC – granule cell, GCL – granule cell layer, GIRK – G-protein-coupled inwardly rectifying potassium channels, GL – glomerular layer, Glu – glutamate, GluR—glutamate receptor, INT – inhibitory interneuron, IPL – internal plexiform layer, K⁺ – potassium, LOT – lateral olfactory tract, MC – mitral cell, MCL – mitral cell layer, ONL – olfactory nerve layer, OSN – olfactory sensory neuron, PG – periglomerular cell, PKA – protein kinase A, PKC – protein kinase C. Black ellipses – speculated excitatory-inhibitory network generating HFO. Receptor images adapted from NIAID Visual & Medical Arts (2024): bioart.niaid.nih.gov/bioart/430 and bioart.niaid.nih.gov/bioart/439

In the current study using local infusion of the D2R agonist quinpirole, directly to the OB, we found a short lasting (around 10 min.) reduction of HFO power followed by a reduction of HFO frequency which persisted to the end of the recording. Speculatively there are several mechanisms that may account for these effects. Electron microscopy and in situ hybridization/radioautography studies have shown D2R to be present on olfactory sensory neuron (OSN) terminals and the apical dendrites of mitral cells (Coronas et al. 1997; Gutiérrez-Mecinas et al. 2005) (Fig. 6). D2R stimulation at OSN terminals may reduce glutamate release (Berkowicz et al. 1994; Ennis et al. 2001) by: 1) reducing cyclic AMP levels, which reduces PKA activity, leading to decreased Ca^2^⁺ channel activity (Wachowiak and Cohen 1999) and 2) enhancing K⁺ efflux through GIRK channels, leading to hyperpolarization (Beaulieu and Gainetdinov 2011). D2R can also directly reduce mitral cells excitability (Hsia et al. 1999) for example by enhancing PKC-mediated phosphorylation of GABA-A receptors, promoting chloride (Cl⁻) influx, causing membrane hyperpolarization (Brünig et al. 1999). As in the OSN, D2R on mitral cells also reduce PKA activity leading to decreased Ca^2^⁺ influx and promote K⁺ efflux through GIRK channels (Ponce et al. 1996) all of which could lead to hyperpolarization and reduced mitral cells firing.

Whilst D2R-mediated reductions in HFO power may be explained by short-lasting effects on excitatory transmission, reductions in HFO frequency are likely to be more complex. D2R activation can affect the kinetics of ion channels in many neuronal subtypes (Benardo and Prince 1982; Lacey et al. 1987; Maurice et al. 2004; Valdés-Baizabal et al. 2015). Slowing of potassium channels, by extending the refractory period of action potentials in mitral/tufted cells, would likely slow the HFO network, potentially contributing to the reduction in HFO frequency observed.

Our finding that stimulation, but not blockade of D2R, affects the HFO rhythm would indirectly suggest that baseline endogenous DA release in the OB is relatively low since the antagonist had no observable effect. However, the agonist quinpirole would still activate D2R and produce the reduction in frequency we observed. DA release in the OB is phasic rather than tonic (Pignatelli and Belluzzi 2017), and as such DA is considered to modulate olfactory sensory input by increasing odour discrimination through mostly inhibitory synaptic mechanisms (Korshunov et al. 2020).

### Differential effects of antipsychotic drugs on HFO frequency

First- and second-generation antipsychotic drugs, both of which act on D2R, differentially affect HFO frequency following NMDAR blockade (Olszewski et al. 2013; Goda et al. 2015; Hunt et al. 2015; Delgado-Sallent et al. 2022; Stan et al. 2024). In the present study, we found that the second-generation antipsychotic drug risperidone reduced MK801-enhanced HFO frequency in the OB (see Fig. 5H), consistent with previous findings in other brain regions (Olszewski et al. 2013; Delgado-Sallent et al. 2022). In contrast, the more selective D2R antagonist eticlopride had no significant effect on HFO frequency (Fig. 4F). In line with this, Stan and colleagues also reported that a selective D2R antagonist, mesdopetam (anti-parkinsonian drug), had no effect on MK801-enhanced HFO (Stan et al. 2024). Considering that risperidone acts on multiple receptors, including D2R, these findings suggest that the reduction in HFO frequency is likely mediated through mechanisms independent of D2R antagonism.

We also investigated aripiprazole, a prototypical third-generation antipsychotic (Mailman and Murthy 2010). This relatively new class of antipsychotics is believed to be more effective in treating negative and cognitive symptoms and has a unique pharmacological profile acting as a DA stabiliser through D2R partial agonism (Bymaster et al. 1996; Wood et al. 2006). We found aripiprazole did not affect HFO frequency (Fig. 5H). This is possibly because when aripiprazole binds to D2R, it elicits a weaker intracellular response than DA itself (Hirose and Kikuchi 2005; Tuplin and Holahan 2017) in contrast to full DA receptor agonists, such as quinpirole and apomorphine, which have a higher affinity and longer duration of action (Durdagi et al. 2016) (see Fig. 2 C, G). Together with previous studies these findings suggest that second, but not first and third-generation antipsychotics, reduce HFO frequency.

### Role of DA in the OB in hyperactivity produced by NMDAR antagonists

Although increase in HFO power and LMA are often associated (Ye et al. 2018; Zepeda et al. 2022; Cui et al. 2022), HFO is not functionally related to movement (Hunt et al. 2006; Wróbel et al. 2020). Indeed, as shown here and in other studies (Olszewski et al. 2013; Hansen et al. 2019) changes in HFO and movement can be dissociated. Further, we showed recently that muscimol inhibition of the OB attenuated MK801-enhanced HFO without affecting hyperactivity (Wróbel et al. 2024). Instead, the HFO rhythm, both at baseline and following NMDAR blockade, appears functionally linked to nasal respiration and sniffing (Wróbel et al. 2020). Thus, while the HFO rhythm generated by the OB is not directly related to LMA, the OB itself appears to have an important role in LMA.

Olfactory bulbectomy (OBX) rats exhibit locomotor hyperactivity, and also display increased LMA after NMDAR antagonists although at a reduced intensity (Redmond et al. 1997; Robichaud et al. 2001). This suggests that OB-related networks can mediate motor control, at least in part. Hyperactivity following bulbectomy may result from compensatory mechanisms for the loss of sensory information leading to changes in exploratory behaviour, LMA, and stress following the loss of a key sensory system (Kelly et al. 1997; Morales-Medina et al. 2017). In our study we found reductions in MK801-induced LMA, following intra OB or systemic injection of DA antagonists, were quantitatively and qualitatively different. For example, we did not observe continuous catalepsy following local infusion as was typical following systemic injection (Fig. 5 F, I). DA signalling in the OB may influence LMA indirectly through broader neural circuits, particularly those related to motivation, reward, and sensory processing (Bromberg-Martin et al. 2010). Since rodents heavily depend on olfaction for exploration (Schultz and Tapp 1973), impaired olfactory processing at D1R and D2R (Escanilla et al. 2009) may underlie the reduced LMA output following MK801 injection.

## Data Availability

The datasets used and analysed during the current study are available from the corresponding author and on RepOD repository at the following link: 10.18150/N97AU1

## References

[CR1] Adraoui FW, Hettak K, Viardot G et al (2024) Differential Effects of Aripiprazole on Electroencephalography-Recorded Gamma-Band Auditory Steady-State Response, Spontaneous Gamma Oscillations and Behavior in a Schizophrenia Rat Model. Int J Mol Sci 25(2):1035. 10.3390/ijms25021035 38256109 10.3390/ijms25021035 PMC10815955

[CR2] Ait Bentaleb K, Boisvert M, Tourjman V, Potvin S (2024) A Meta-Analysis of Functional Neuroimaging Studies of Ketamine Administration in Healthy Volunteers. J Psychoactive Drugs 56(2):211–224. 10.1080/02791072.2023.2190758 36921026 10.1080/02791072.2023.2190758

[CR3] Beaulieu JM, Gainetdinov RR (2011) The physiology, signaling, and pharmacology of dopamine receptors. Pharmacol Rev 63(1):182–217. 10.1124/pr.110.002642 21303898 10.1124/pr.110.002642

[CR4] Benardo and Prince (1982) Dopamine modulates a Ca2+ -activated potassium conductance in mammalian hippocampal pyramidal cells. Nature 297(5861):76–9. 10.1038/297076a0 6280074 10.1038/297076a0

[CR5] Benes FM (2000) Emerging principles of altered neural circuitry in schizophrenia 1. Brain Res Rev. 31(2–3):251–69. 10.1016/s0165-0173(99)00041-7 10719152 10.1016/s0165-0173(99)00041-7

[CR6] Berkowicz DA, Trombley PQ, Shepherd GM (1994) Evidence for glutamate as the olfactory receptor cell neurotransmitter. J Neurophysiol. 71(6):2557–61. 10.1152/jn.1994.71.6.2557 7931535 10.1152/jn.1994.71.6.2557

[CR7] Bianciardi B, Uhlhaas PJ (2021) Do NMDA-R antagonists re-create patterns of spontaneous gamma-band activity in schizophrenia? A systematic review and perspective. Neurosci Biobehav Rev 124:308–323. 10.1016/j.neubiorev.2021.02.005 33581223 10.1016/j.neubiorev.2021.02.005

[CR8] Björklund A, Dunnett SB (2007) Dopamine neuron systems in the brain: an update. Trends Neurosci 30(5):194–202. 10.1016/j.tins.2007.03.006 17408759 10.1016/j.tins.2007.03.006

[CR9] Bromberg-Martin ES, Matsumoto M, Hikosaka O (2010) Dopamine in Motivational Control: Rewarding, Aversive, and Alerting. Neuron 68(5):815–34. 10.1016/j.neuron.2010.11.022 21144997 10.1016/j.neuron.2010.11.022 PMC3032992

[CR10] Brünig I, Sommer M, Hatt H, Bormann J (1999) Dopamine receptor subtypes modulate olfactory bulb gamma-aminobutyric acid type A receptors. Proc National Acad Sci USA 96(5):2456–60. 10.1073/pnas.96.5.2456 10.1073/pnas.96.5.2456 PMC26806 10051664

[CR11] Brys I, Barrientos SA, Ward JE et al (2023) 5-HT2AR and NMDAR psychedelics induce similar hyper-synchronous states in the rat cognitive-limbic cortex-basal ganglia system. Communications Biology. Nature Res 6(1):737. 10.1038/s42003-023-05093-6 10.1038/s42003-023-05093-6 PMC10372079 37495733

[CR12] Buzsáki G, Logothetis N, Singer W (2013) Scaling brain size, keeping timing: Evolutionary preservation of brain rhythms. Neuron 80(3):751–64. 10.1016/j.neuron.2013.10.002 24183025 10.1016/j.neuron.2013.10.002 PMC4009705

[CR13] Bymaster FP, Calligaro D, Falcone JF et al (1996) Radioreceptor Binding Profile of the Atypical Antipsychotic Olanzapine. Neuropsychopharmacology 14(2):87–96. 10.1016/0893-133X(94)00129-N 8822531 10.1016/0893-133X(94)00129-N

[CR14] Castner SA, Williams GV (2007) Tuning the engine of cognition: A focus on NMDA/D1 receptor interactions in prefrontal cortex. Brain Cogn 63:94–122. 10.1016/j.bandc.2006.11.002 17204357 10.1016/j.bandc.2006.11.002

[CR15] Castro-Zaballa S, González J, Cavelli M, Mateos D, Pascovich C, Tort A, Hunt MJ, Torterolo P (2025) Cortical high-frequency oscillations (≈110 Hz) in cats are state-dependent and enhanced by a subanesthetic dose of ketamine. Behav Brain Res 476:115231. 10.1016/j.bbr.2024.115231 10.1016/j.bbr.2024.115231 39218075

[CR16] Chen YN, Kostka JK, Bitzenhofer SH, Hanganu-Opatz IL (2023) Olfactory bulb activity shapes the development of entorhinal-hippocampal coupling and associated cognitive abilities. Curr Biol 33:4353-4366.e5. 10.1016/j.cub.2023.08.072 37729915 10.1016/j.cub.2023.08.072 PMC10617757

[CR17] Coronas V, Srivastava LK, Liang JJ, Jourdan F, Moyse E (1997) Identification and localization of dopamine receptor subtypes in rat olfactory mucosa and bulb: a combined in situ hybridization and ligand binding radioautographic approach. J Chem Neuroanat 12(4):243–57. 10.1016/s0891-0618(97)00215-9 9243344 10.1016/s0891-0618(97)00215-9

[CR18] Cui K, Yu Z, Xu L et al (2022) Behavioral features and disorganization of oscillatory activity in C57BL/6J mice after acute low dose MK-801 administration. Front Neurosci 16:1001869. 10.3389/fnins.2022.1001869 36188453 10.3389/fnins.2022.1001869 PMC9515662

[CR19] Delgado-Sallent C, Nebot P, Gener T et al (2022) Phencyclidine-induced psychosis causes hypersynchronization and disruption of connectivity within prefrontal-hippocampal circuits that is rescued by antipsychotic drugs. Cereb Cortex 32(16):3472–3487. 10.1093/cercor/bhab427 34875009 10.1093/cercor/bhab427

[CR20] Dunah AW, Standaert DG (2001) Dopamine D1 receptor-dependent trafficking of striatal NMDA glutamate receptors to the postsynaptic membrane. J Neurosci 21(15):5546–5558. 10.1523/JNEUROSCI.21-15-05546.2001 11466426 10.1523/JNEUROSCI.21-15-05546.2001 PMC6762635

[CR21] Durdagi S, Salmas RE, Stein M et al (2016) Binding Interactions of Dopamine and Apomorphine in D2High and D2Low States of Human Dopamine D2 Receptor Using Computational and Experimental Techniques. ACS Chem Neurosci 7(2):185–95. 10.1021/acschemneuro.5b00271 26645629 10.1021/acschemneuro.5b00271

[CR22] Ennis M, Zhou F-M, Ciombor KJ et al (2001) Dopamine D2 Receptor-Mediated Presynaptic Inhibition of Olfactory Nerve Terminals. J Neurophysiol 86(6):2986–97. 10.1152/jn.2001.86.6.2986 11731555 10.1152/jn.2001.86.6.2986

[CR23] Escanilla O, Yuhas C, Marzan D et al (2009) Dopaminergic Modulation of Olfactory Bulb Processing Affects Odor Discrimination Learning in Rats. Behav Neurosci 123(4):828–33. 10.1037/a0015855 19634942 10.1037/a0015855 PMC2766664

[CR24] Faraone SV (2018) The pharmacology of amphetamine and methylphenidate: Relevance to the neurobiology of attention-deficit/hyperactivity disorder and other psychiatric comorbidities. Neurosci Biobehav Rev. Apr;87:255–270. 10.1016/j.neubiorev.2018.02.001. 10.1016/j.neubiorev.2018.02.001 PMC8063758 29428394

[CR25] Fischer T, Scheffler P, Lohr C (2020) Dopamine-induced calcium signaling in olfactory bulb astrocytes. Sci Rep 10(1):631. 10.1038/s41598-020-57462-4 10.1038/s41598-020-57462-4 PMC6971274 31959788

[CR26] Goda SA, Olszewski M, Piasecka J et al (2015) Aberrant high frequency oscillations recorded in the rat nucleus accumbens in the methylazoxymethanol acetate neurodevelopmental model of schizophrenia. Progress in Neuro-Psychopharmacology and Biological Psychiatry 61:44–51. 10.1016/j.pnpbp.2015.03.016 25862088 10.1016/j.pnpbp.2015.03.016

[CR27] Gutièrrez-Mecinas M, Crespo C, Blasco-Ibáñez JM et al (2005) Distribution of D2 dopamine receptor in the olfactory glomeruli of the rat olfactory bulb. Eur J Neurosci 22(6):1357–67. 10.1111/j.1460-9568.2005.04328.x 16190891 10.1111/j.1460-9568.2005.04328.x

[CR28] Hakami T, Jones NC, Tolmacheva EA, et al. (2009) NMDA receptor hypofunction leads to generalized and persistent aberrant γ oscillations independent of hyperlocomotion and the state of consciousness. *PLoS ONE* 4(8):e6755. 10.1371/journal.pone.0006755. 10.1371/journal.pone.0006755 PMC2727800 19707548

[CR29] Hansen IH, Agerskov C, Arvastson L, Bastlund JF, Sørensen HBD, Herrik KF (2019) Pharmaco-electroencephalographic responses in the rat differ between active and inactive locomotor states. Eur J Neurosci 50(2):1948–1971. 10.1111/ejn.14373 30762918 10.1111/ejn.14373 PMC6806018

[CR30] Hirose and Kikuchi (2005) Aripiprazole, a novel antipsychotic agent: dopamine D2 receptor partial agonist. J Med Invest 52 suppl:284–290. 10.2152/jmi.52.284 16366516 10.2152/jmi.52.284

[CR31] Hiyoshi T, Kambe D, Karasawa JI et al (2014) Differential effects of NMDA receptor antagonists at lower and higher doses on basal gamma band oscillation power in rat cortical electroencephalograms. Neuropharmacology 85:384–96. 10.1016/j.neuropharm.2014.05.037 24907590 10.1016/j.neuropharm.2014.05.037

[CR32] Homayoun H, Moghaddam B (2007) NMDA receptor hypofunction produces opposite effects on prefrontal cortex interneurons and pyramidal neurons. J Neurosci 27:11496–11500. 10.1523/JNEUROSCI.2213-07.2007 17959792 10.1523/JNEUROSCI.2213-07.2007 PMC2954603

[CR33] Hsia AY, Vincent JD and Lledo PM (1999) Dopamine Depresses Synaptic Inputs Into the Olfactory Bulb. 82(2):1082–5. 10.1152/jn.1999.82.2.1082. 10.1152/jn.1999.82.2.1082 10444702

[CR34] Hunt MJ, Kasicki S (2013) A systematic review of the effects of NMDA receptor antagonists on oscillatory activity recorded in vivo. J Psychopharmacol 27(11):972–86. 10.1177/0269881113495117 23863924 10.1177/0269881113495117

[CR35] Hunt MJ, Raynaud B, Garcia R (2006) Ketamine Dose-Dependently Induces High-Frequency Oscillations in the Nucleus Accumbens in Freely Moving Rats. Biol Psychiatr 60(11):1206–14. 10.1016/j.biopsych.2006.01.020 10.1016/j.biopsych.2006.01.020 16650831

[CR36] Hunt MJ, Falinska M, Kasicki S (2010) Local injection of MK801 modifies oscillatory activity in the nucleus accumbens in awake rats. J Psychopharmacol 24:931–941. 10.1177/0269881109102539 19329548 10.1177/0269881109102539

[CR37] Hunt MJ, Olszewski M, Piasecka J et al (2015) Effects of NMDA receptor antagonists and antipsychotics on high frequency oscillations recorded in the nucleus accumbens of freely moving mice. Psychopharmacol 164(2):380–6. 10.1016/j.neuroscience.2009.08.047 10.1007/s00213-015-4073-0 PMC4646921 26446869

[CR38] Hunt MJ, Adams NE, Średniawa W et al (2019) The olfactory bulb is a source of high-frequency oscillations (130–180 Hz) associated with a subanesthetic dose of ketamine in rodents. Neuropsychopharmacol 44(2):435–442. 10.1038/s41386-018-0173-y 10.1038/s41386-018-0173-y PMC6300534 30140046

[CR39] Jenner P, Katzenschlager R (2016) Apomorphine—pharmacological properties and clinical trials in Parkinson’s disease. Parkinsonism Relat Disord 33 Suppl 1:S13–S21. 10.1016/j.parkreldis.2016.12.003 10.1016/j.parkreldis.2016.12.003 27979722

[CR40] Kelly JP, Wrynn AS, Leonard BE (1997) The olfactory bulbectomized rat as a model of depression: an update. Pharmacol Ther 74(3):299–316. 10.1016/s0163-7258(97)00004-1 9352586 10.1016/s0163-7258(97)00004-1

[CR41] Korshunov KS, Blakemore LJ, Bertram R, Trombley PQ (2020) Spiking and Membrane Properties of Rat Olfactory Bulb Dopamine Neurons. Front Cell Neurosci 14:60. 10.3389/fncel.2020.00060 32265662 10.3389/fncel.2020.00060 PMC7100387

[CR42] Lacey MG, Mercurit NB, North RA (1987) Dopamine acts on D2 receptors to increase potassium conductance in neurones of the rat substantia nigra zona compacta. J Physiol 392:397–416. 10.1113/jphysiol.1987.sp016787 2451725 10.1113/jphysiol.1987.sp016787 PMC1192311

[CR43] Lee DK, Ahn SM, Shim Y-B, Koh WCA, Shim I, Choe ES (2011) Interactions of Dopamine D1 and N-methyl-D-Aspartate Receptors Are Required for Acute Cocaine-Evoked Nitric Oxide Efflux in the Dorsal Striatum. Exp Neurobiol 20:116–122. 10.5607/en.2011.20.2.116 22110369 10.5607/en.2011.20.2.116 PMC3213699

[CR44] Lee J, Hudson MR, O’Brien TJ et al (2017) Local NMDA receptor hypofunction evokes generalized effects on gamma and high-frequency oscillations and behavior. Neurosci 358:124–136. 10.1016/j.neuroscience.2017.06.039 10.1016/j.neuroscience.2017.06.039 28676240

[CR45] Lisman JE, Coyle JT, Green RW, Javitt DC, Benes FM, Heckers S, Grace AA (2008) Circuit-based framework for understanding neurotransmitter and risk gene interactions in schizophrenia. Trends Neurosci 31:234–242. 10.1016/j.tins.2008.02.005 18395805 10.1016/j.tins.2008.02.005 PMC2680493

[CR46] Liu S (2020) Dopaminergic Modulation of Glomerular Circuits in the Mouse Olfactory Bulb. Front Cell Neurosci Jun 12;14:172. 10.3389/fncel.2020.00172. 10.3389/fncel.2020.00172 PMC7304284 32595457

[CR47] Lothmann K, Amunts K, Herold C (2021) The neurotransmitter receptor architecture of the mouse olfactory system. Front Neuroanat 15:632549. 10.3389/fnana.2021.632549 10.3389/fnana.2021.632549 PMC8102831 33967704

[CR48] Mailman RB, Murthy V (2010) (2010) Third generation antipsychotic drugs: partial agonism or receptor functional selectivity? Curr Pharm des 16(5):488–501. 10.2174/138161210790361461 19909227 10.2174/138161210790361461 PMC2958217

[CR49] Matulewicz P, Kasicki S and Hunt MJ (2010) The effect of dopamine receptor blockade in the rodent nucleus accumbens on local field potential oscillations and motor activity in response to ketamine. Brain Res 1366:226–32. 10.1016/j.brainres.2010.09.088. 10.1016/j.brainres.2010.09.088 20888326

[CR50] Maurice N, Mercer J, Chan CS et al (2004) D2 dopamine receptor-mediated modulation of voltage-dependent Na+ channels reduces autonomous activity in striatal cholinergic interneurons. J Neurosci 24(46):10289–301. 10.1523/JNEUROSCI.2155-04.2004 15548642 10.1523/JNEUROSCI.2155-04.2004 PMC6730305

[CR51] Morales-Medina JC, Iannitti T, Freeman A, Caldwell HK (2017) The olfactory bulbectomized rat as a model of depression: The hippocampal pathway. Behav Brain Res 317:562–575. 10.1016/j.bbr.2016.09.029 27633561 10.1016/j.bbr.2016.09.029

[CR52] NIAID Visual & Medical Arts. Receptor protein [Internet]. NIAID NIH BIOART Source; 2024 Oct 7 [cited 2025 Apr 5]. Available from: https://bioart.niaid.nih.gov/bioart/430

[CR53] NIAID Visual & Medical Arts. Receptor protein [Internet]. NIAID NIH BIOART Source; 2024 Oct 7 [cited 2025 Apr 5]. Available from: https://bioart.niaid.nih.gov/bioart/439

[CR54] Nottage JF, Gabay A, De Meyer K et al (2023) The effect of ketamine and D-cycloserine on the high frequency resting EEG spectrum in humans. Psychopharmacology 240(1):59–75. 10.1007/s00213-022-06272-9 36401646 10.1007/s00213-022-06272-9 PMC9816261

[CR55] Nugent AC, Ballard ED, Gould TD, Park LT, Moaddel R, Brutsche NE, Zarate CA Jr (2019) Ketamine has distinct electrophysiological and behavioral effects in depressed and healthy subjects. Mol Psychiatry 24(7):1040–1052. 10.1038/s41380-018-0028-2 10.1038/s41380-018-0028-2 PMC6111001 29487402

[CR58] Olszewski M, Piasecka J, Goda SA, Kasicki S, Hunt MJ (2013) Antipsychotic compounds differentially modulate high-frequency oscillations in the rat nucleus accumbens: a comparison of first- and second-generation drugs. Int J Neuropsychopharmacol 16(5):1009–1020. 10.1017/S1461145712001034 23171738 10.1017/S1461145712001034

[CR56] Olney JW, Labruyere J, Wang G, Wozniak DF, Price MT, Sesma MA (1991) NMDA antagonist neurotoxicity: mechanism and prevention. Science 254:1515–1518. 10.1126/science.1835799 10.1126/science.1835799 1835799

[CR57] Olney JW, Newcomer JW, Farber NB (1999) NMDA receptor hypofunction model of schizophrenia. J Psychiatr Res Nov-Dec 33(6):523–533. 10.1016/s0022-3956(99)00029-1 10.1016/s0022-3956(99)00029-1 10628529

[CR60] Ouagazzal A, Amalric M (1995) Competitive NMDA receptor antagonists do not produce locomotor hyperactivity by a dopamine-dependent mechanism. Eur J Pharmacol 294(1):137–146. 10.1016/0014-2999(95)00518-8.PMID:8788425.(n.d.) 8788425 10.1016/0014-2999(95)00518-8

[CR61] Pignatelli A, Belluzzi O (2017) Dopaminergic neurones in the main olfactory bulb: An overview from an electrophysiological perspective. Front Neuroanat 11:7. 10.3389/fnana.2017.00007 28261065 10.3389/fnana.2017.00007 PMC5306133

[CR62] Ponce A, Bueno E, Kentros C, Vega-Saenz de Miera E, Chow A, Hillman D, Chen S, Zhu L, Wu MB, Wu X, Rudy B, Thornhill WB (1996) G-protein-gated inward rectifier K+ channel proteins (GIRK1) are present in the soma and dendrites as well as in nerve terminals of specific neurons in the brain. J Neurosci. 16(6):1990–2001. 10.1523/JNEUROSCI.16-06-01990.1996 8604043 10.1523/JNEUROSCI.16-06-01990.1996 PMC6578514

[CR63] Redmond Anna M, Kelly John P, Leonard BE et al (1997) Behavioural and Neurochemical Effects of Dizocilpine in the Olfactory Bulbectomized Rat Model of Depression. PharmacolBiochem Behav. 58(2):355–9. 10.1016/s0091-3057(97)00259-1 10.1016/s0091-3057(97)00259-1 9300592

[CR64] Robichaud M, Beauchemin V, Lavoie N et al (2001) Effects of bilateral olfactory bulbectomy on N-methyl-D-aspartate receptor function: Autoradiographic and behavioral studies in the rat. Synapse 42(2):95–103. 10.1002/syn.1105 11574946 10.1002/syn.1105

[CR65] Schoppa NE (1998) Dendrodendritic inhibition in the olfactory bulb is driven by NMDA receptors. J Neurosci 18(17):6790–802. 10.1523/JNEUROSCI.18-17-06790.1998 9712650 10.1523/JNEUROSCI.18-17-06790.1998 PMC6792983

[CR66] Schultz EF, Tapp JT (1973) Olfactory control of behavior in rodents. 1, Psychological Bulletin, 79(1), 21–44. 10.1037/h0033817 10.1037/h0033817 4567728

[CR67] Sokolenko E, Hudson MR, Nithianantharajah J et al (2019) The mGluR2/3 agonist LY379268 reverses NMDA receptor antagonist effects on cortical gamma oscillations and phase coherence, but not working memory impairments, in mice. J Psychopharmacol 33(12):1588–1599. 10.1177/0269881119875976 31580222 10.1177/0269881119875976

[CR68] Speers LJ, Bilkey DK (2021) Disorganization of oscillatory activity in animal models of schizophrenia. Front Neural Circ 15:741767. 10.3389/fncir.2021.741767 10.3389/fncir.2021.741767 PMC8523827 34675780

[CR69] Średniawa W, Wróbel J, Kublik E et al (2021) Network and synaptic mechanisms underlying high frequency oscillations in the rat and cat olfactory bulb under ketamine-xylazine anesthesia. Sci Rep 11(1):6390. 10.1038/s41598-021-85705-5 33737621 10.1038/s41598-021-85705-5 PMC7973548

[CR70] Stan TL, Ronaghi A, Barrientos SA, et al. (2024) Neurophysiological treatment effects of mesdopetam, pimavanserin and clozapine in a rodent model of Parkinson’s disease psychosis. *Neurotherapeutics* 21(2). Mar;21(2):e00334. 10.1016/j.neurot.2024.e00334. 10.1016/j.neurot.2024.e00334 PMC10937958 38368170

[CR71] Su YA, Si TM, Zhou DF et al (2007) Risperidone attenuates MK-801-induced hyperlocomotion in mice via the blockade of serotonin 5-HT2A/2C receptors. Eur J Pharmacol 564(1–3):123–30. 10.1016/j.ejphar.2007.02.031 17395179 10.1016/j.ejphar.2007.02.031

[CR72] Trimper JB, Galloway CR, Jones AC, Mandi K, Manns JR (2017) Gamma Oscillations in Rat Hippocampal Subregions Dentate Gyrus, CA3, CA1, and Subiculum Underlie Associative Memory Encoding. Cell Rep 21:2419–2432. 10.1016/j.celrep.2017.10.123 29186681 10.1016/j.celrep.2017.10.123 PMC5728687

[CR73] Tuplin EW, Holahan MR (2017) Aripiprazole, A Drug that Displays Partial Agonism and Functional Selectivity. Current Neuropharmacol 15(8):1192–1207. 10.2174/1570159X15666170413115754 10.2174/1570159X15666170413115754 PMC5725548 28412910

[CR74] Valdés-Baizabal C, Soto E and Vega R (2015) Dopaminergic modulation of the voltage-gated sodium current in the cochlear afferent neurons of the rat. PLoS ONE 10(3):e0120808. 10.1371/journal.pone.0120808. 10.1371/journal.pone.0120808 PMC4359166 25768433

[CR75] Wachowiak M, Cohen LB. (1999) Presynaptic inhibition of primary olfactory afferents mediated by different mechanisms in lobster and turtle. J Neurosci*.* 19(20):8808–17. 10.1523/JNEUROSCI.19-20-08808.1999. 10.1523/JNEUROSCI.19-20-08808.1999 PMC6782745 10516300

[CR76] Williams GV, Castner SA (2006) Under the curve: Critical issues for elucidating D1 receptor function in working memory. Neuroscience 139:263–276. 10.1016/j.neuroscience.2005.09.028 16310964 10.1016/j.neuroscience.2005.09.028

[CR77] Wood MD, Scott C, Clarke K, Cato KJ, Patel N, Heath J, Worby A, Gordon L, Campbell L, Riley G, Davies CH, Gribble A, Jones DNC (2006) Pharmacological Profile of Antipsychotics at Monoamine Receptors: Atypicality Beyond 5-HT 2A Receptor Blockade. CNS & Neurological Disorders-Drug Targets. 5(4):445–52. 10.2174/187152706777950693 16918396 10.2174/187152706777950693

[CR78] Wróbel J, Średniawa W, Jurkiewicz G et al (2020) Nasal respiration is necessary for ketamine-dependent high frequency network oscillations and behavioral hyperactivity in rats. Sci Rep 10(1):18981. 10.1038/s41598-020-75641-1 33149202 10.1038/s41598-020-75641-1 PMC7642442

[CR79] Wróbel J, Średniawa W, Bramorska A, Dovgialo M, Wójcik DK, Hunt MJ (2024) NMDA receptor antagonist high-frequency oscillations are transmitted via bottom-up feedforward processing. Sci Rep 14(1):21858. 10.1038/s41598-024-71749-w 10.1038/s41598-024-71749-w PMC11413191 39300126

[CR80] Yamaguchi T, Goto A, Nakahara I, Yawata S, Hikida T, Matsuda M, Funabiki K, Nakanishi S (2015) Role of PKA signaling in D2 receptor-expressing neurons in the core of the nucleus accumbens in aversive learning. Proc National Acad Sci USA 112(36):11383–11388. 10.1073/pnas.1514731112 10.1073/pnas.1514731112 PMC4568655 26305972

[CR81] Yan T, Suzuki K, Kameda S, Maeda M, Mihara T, Hirata M. (2022) Electrocorticographic effects of acute ketamine on non-human primate brains. J Neural Eng 19(2). 10.1088/1741-2552/ac6293. 10.1088/1741-2552/ac6293 35354131

[CR82] Ye T, Bartlett MJ, Schmit MB et al (2018) Ten-Hour Exposure to Low-Dose Ketamine Enhances Corticostriatal Cross-Frequency Coupling and Hippocampal Broad-Band Gamma Oscillations. Front Neural Circ 12:61. 10.3389/fncir.2018.00061 10.3389/fncir.2018.00061 PMC6099120 30150926

[CR83] Zepeda NC, Crown LM, Medvidovic S, Choi W, Sheth M, Bergosh M, Gifford R, Folz C, Lam P, Lu G, Featherstone R, Liu CY, Siegel SJ, Lee DJ (2022) Frequency-specific medial septal nucleus deep brain stimulation improves spatial memory in MK-801-treated male rats. Neurobiol Dis 170:105756. 10.1016/j.nbd.2022.105756 10.1016/j.nbd.2022.105756 PMC9343054 35584727

